# Tunable Lotus Leaf Effect by Three-Dimensionally Printed Stretchable Objects

**DOI:** 10.1021/acsami.4c14238

**Published:** 2024-11-06

**Authors:** Noa Trink, Shlomo Magdassi

**Affiliations:** a Institute of Chemistry and The Center for Nanoscience and Nanotechnology, The Hebrew University of Jerusalem, Jerusalem 9190401, Israel; b Singapore-HUJ Alliance for Research and Enterprise (SHARE), Smart Grippers for Soft Robotics (SGSR), Campus for Research Excellence and Technological Enterprise (CREATE), Singapore138602, Singapore

**Keywords:** superhydrophobicity, 3D printing, soft materials, particles, tuned wettability

## Abstract

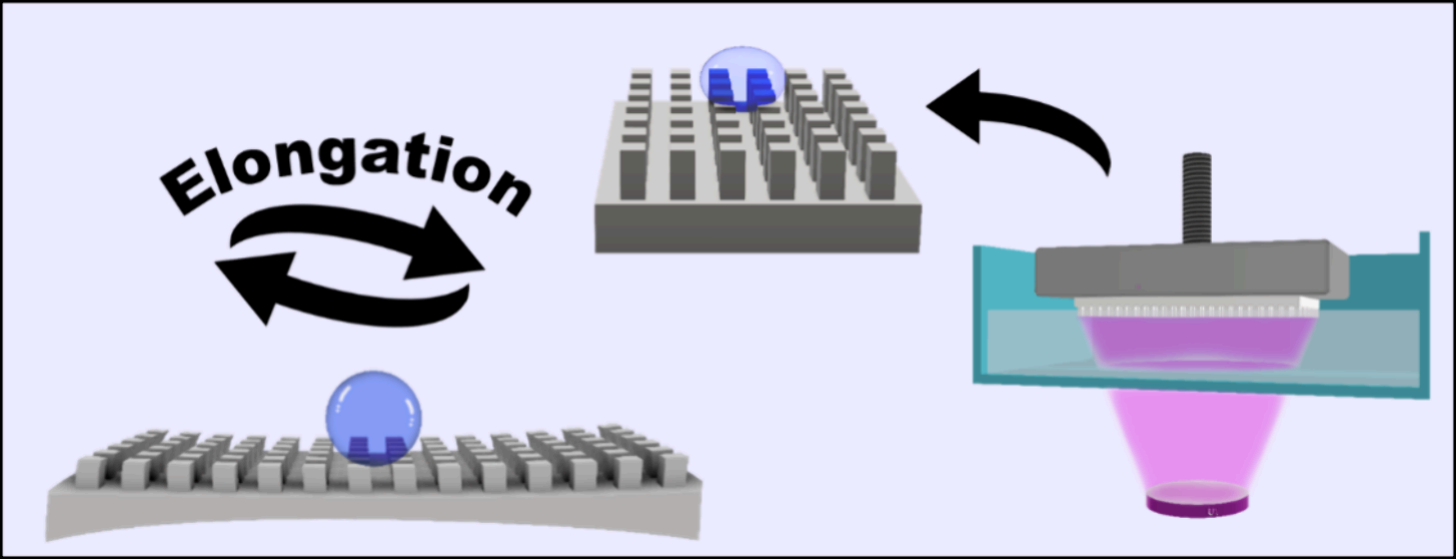

Adjustable wettability is important for various fields, such as droplet manipulation and controlled surface adhesion. Herein, we present high-resolution 3D stretchable structures with tunable superhydrophobicity, fabricated by a stereolithography-based printing process. The printing compositions comprise nonfluorinated monomers based on silicone urethane with dispersed hydrophobic silica particles. 3D lotus-like structures were designed and printed, having microsize pillars located at the external surfaces, with controlled dimensions and interspacing. The design of the pillars and the presence of the hydrophobic silica particles resulted in superhydrophobicity due to the surface structuring and entrapment of air between the pillars. The best structures display a contact angle of 153.3° ± 1.3° and rolling angle of 3.3° ± 0.5°, and their self-cleaning, water repellency, and buoyancy are demonstrated. The durability of the structure over time, water immersion, and heat exposure were tested, confirming the preservation of superhydrophobicity under these conditions. Upon stretching the surfaces, the interpillar distances change, thus enabling tuning the wetting properties and achieving good control over the contact and rolling angles, while the stretching-induced superhydrophobicity is reversible. This approach can expand the potential applications of superhydrophobic soft materials to fields requiring control over the wetting properties, including soft robotics, biomedical devices, and stretchable electronics.

## 1 Introduction

Superhydrophobicity is characterized by extreme water-repellency and self-cleaning behavior^1−3^ which can be observed in nature among plants and animals, enabling them to stay clean and dry in variable conditions.^4^ The *″Lotus effect*″, which imparts the self-cleaning ability of superhydrophobic structures, was first presented by Barthlott and Neinhuis in 1997.^5^ The key to achieving superhydrophobicity lies in combining hierarchical surface structures and low-surface energy materials.^6^ These surface structuring and roughness, at the nano to microscale range, cause the water drop to appear in Cassie–Baxter state, in which air is trapped between the water droplets and the surface,^7^ resulting in a composite surface. The contact angle can be described by the Cassie–Baxter eq (eq 1).
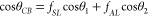
1

θ_CB_ is the contact angle of the Cassie–Baxter state, f_SL_ is the friction of the solid–liquid surface, f_AL_ is the air–liquid surface, θ_1_ is the contact angle of the liquid on a smooth surface and θ_2_ is the contact angle of the liquid with the air. Since θ_2_ = 180°, eq 2 is received.^8,9^
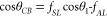
2

It can be noted that a change in f_SL_ will immediately result in a shift in the contact angle, thus providing a method to control it. To qualify a surface as superhydrophobic, it should have a water contact angle exceeding 150° and a rolling angle of less than 10°.^10^ Another known method to characterize a surface as superhydrophobic is low contact angle hysteresis (lower than 10°).^11^ The application of superhydrophobicity is significant in many fields, including marine, aerospace, medical devices, and textiles.^12^ These applications are based on unique properties, including self-cleaning,^13,14^ anti-icing,^15,16^ facilitation of oil–water separation,^17^ corrosion resistance,^18^ and drag reduction.^19^ Conventional approaches to attain superhydrophobicity typically involve combining chemical surface modifications or coatings,^20,21^ fluorinated materials,^22−24^ or polydimethylsiloxane (PDMS)^25−28^ with making surface roughness by etching^29−31^ and molding techniques which enable the creation of micropatterns on the surface.^32−34^ These methods usually require multistep fabrication processes,^35^ and there is an unmet need for a simple fabrication process to create complex functional structures with programmable properties. Fabrication by 3D printing, also known as additive manufacturing (AM), enables the making of highly accurate and complex 3D geometries with customizable microarchitectures.^36,37^ Various printing techniques exist, including Fused Deposition Modeling (FDM),^38,39^ Direct Ink Writing (DIW),^40,41^ and Digital Light Processing (DLP).^42^ Fabrication of superhydrophobic surfaces by AM processes was reported mainly by two-photon polymerization and stereolithography, as presented in several reviews.^43,44^ Recently, Zhao et. al presented an FDM printed polylactic acid microplate, which, upon coating with PDMS and hydrophobic silica particles, resulted in reversible wetting based on heating by utilizing the shape memory effect.^45^ The DLP technique is based on a localized photopolymerization process activated by UV irradiation, using proper monomers and photoinitiators. It can produce a wide range of complex 3D structures with precise microscale architecture and higher printing resolution and complexity than FDM and DIW printing,^46,47^ where resolution is constrained by the nozzle diameter.^48^ In comparison to DLP, FDM can provide a cost-effective option^48^ and is considered the most common printing method.^49^ Despite this, DLP is a relatively faster printing method, as each layer is formed in a single exposure, unlike FDM and DIW, which build structures line-by-line.^50^ Furthermore, in recent years the cost of DLP printers has become low, with some in the cost range of simple FDM printers.^51^ Unlike other reported printing methods for surface structuring of polymers, mainly DIW and FDM that result in continuous structures, DLP printing enables a higher degree of flexibility in design, by obtaining discrete features on the surfaces of complex 3D objects. Furthermore, the hierarchical structure can be obtained by two means; by the structure design, and by the inherent feature of the internal layering of the DLP layer-by-layer printing process. This, combined with the light-induced localized polymerization, enables precise control of the surface structure of 3D objects.

In a previous report, we demonstrated the direct fabrication of superhydrophobic 3D rigid objects by DLP printing, eliminating the need for any surface modification to achieve superhydrophobicity.^52^ Although the fabrication of superhydrophobic surfaces by printing was reported,^53^ yet limited attention has been directed toward making objects with tunable and controllable wetting properties by the 3D-printing method. Our current study aims to develop complex stretchable 3D structures with tunable superhydrophobicity and self-cleaning properties based on photopolymerizable soft materials. Combining complex shapes with material deformation upon stretching, together with hydrophobic particles that bring a submicron surface roughness, enables simple tuning of wetting properties, which has not been reported yet. The tuning of the reversible wetting is achieved simply by stretching the elastic 3D-printed structure. To achieve this, we have developed new ink compositions that result in stretchable polymers, composed of nonfluorinated acrylic monomers based on silicone urethane acrylate (SUA) with dispersed hydrophobic fumed silica (HFS) particles. Using the DLP method, an array of microscale pillars was printed, while the chemical composition (embedded hydrophobic nanoparticles) and the structural design (printed micropillars) resulted in superhydrophobic objects. Control over the wetting properties was previously reported by using PDMS with roughness created by laser ablation^54^ photolithography^55^ or molding.^56−59^ Unlike reported approaches to achieve control of wettability by stretching, mostly based on molding, controlling surface wetting through 3D printing techniques, such as DLP, opens up new possibilities for creating complex and functional devices with preprogrammed and controllable surface-wetting properties, in a single fabrication step. This capability makes it possible to fabricate complex functional devices, such as soft grippers, with tailored wetting properties. The complex structures can be simply fabricated using the printing method, unlike the other methods in which the production process can be long, challenging, and for some structures even impossible. So far, only a few publications have reported controlling wetting properties using 3D-printing techniques, that resulted in low-resolution structures. One recent publication demonstrated programmable anisotropic wettability of a surface through DIW printing.^60^ A stretchable film composed of a soft silicone elastomer was fabricated, having continuous mesh structures, in which the line width is about 200 μm. The contact angle could be controlled by stretching the film, which caused an anisotropic change in the mesh structure. The resulting contact angles of the printed films were in the range of 120°–160°. An interesting application was demonstrated by mounting the film on top of a soft gripper, enabling water-proof that could be controlled by bending. However, the sliding angle of those printed films was large, in the range of 20°- 40°, maybe due to the low inherent resolution and the limited, simple complexity that can be achieved by DIW printing. This current work aims to utilize DLP printing technology and elastomer deformation to overcome the above presented limitations of the other printing techniques and open up the field of tunable superhydrophobicity of soft printed materials to be used in various fields such as soft robotics,^61,62^ wearable and stretchable electronic devices,^63^ and medical equipment.

## 2 Experimental Section

### 2.1 Materials

Difunctional aliphatic silicone urethane acrylate oligomers BRS-14320S (SUA-1) and XRLH-3–142 (SUA-2) were kindly provided by BOMAR (Dymax Europe GmbH, Germany). Monofunctional monomer lauryl acrylate (SR335) was kindly provided by Sartomer-Arkema (Colombes Cedex, France). Photoinitiator diphenyl(2,4,6-trimethylbenzoyl)phosphine oxide (TPO) and isobornyl acrylate (IBOA), were purchased from BASF (Ludwigshafen, Germany) and Alfa Aesar, respectively. HFS, Hydrophobic fumed silica (TS-610), with submicron particles size (0.2–0.3 μm), were kindly provided by Cabot Specialty Chemicals Inc. Sudan III, 1-[4-(phenylazo)phenylazo]-2-naphthol, was purchased from Sigma-Aldrich. For measuring contact and rolling angles double distilled water was used. Figure S1 displays the structure of all reactants and materials, alongside the photopolymerization reaction.

### 2.2 Printing Compositions

For the experiments conducted, 30 g of SUA printing formulation was typically prepared. Using SUA-1 oligomer, the ink was formulated by mixing 5.4 g of SR335 (18 wt %) and 2.4 g of IBOA (8 wt %). Next, 0.6 g of TPO (2 wt %) was added. To obtain a high resolution, 0.002 g of Sudan III (0.01 wt %) was added to some formulations. The mixture was dissolved under bath sonication (Elmasonic P 30H) at 60°*C* with 100% power at 80 kHz for 20 min. Afterward, 20.1 g of SUA-1 (67 wt %) was added under manual mixing. Finally, 1.5 g of HFS particles (5 wt %) were added under additional manual mixing. A second printing composition with enhanced mechanical properties was made by using SUA-2 oligomer. In this mixture, SR335 and XRLH-3–142 were mixed at a 1:1 weight ratio. First, 13.950 g of SR335 (46.5 wt %), 0.600 g of TPO (2 wt %), and 0.002 g of Sudan III (0.01 wt %) were mixed and dissolved under bath sonication using the above parameters. Afterward, 13.950 g of SUA-2 (46.5 wt %) was added under manual mixing. Subsequently, 1.5 g of HFS particles (5 wt %) were added under 5 min mechanical mixing using DISPERMAT CV (Reichshof). Both ink compositions underwent 2 min defoaming process using a Planetary Centrifugal Mixer (Thinky).

### 2.3 DLP 3D Printing

The DLP printing was performed by Asiga Max X35 UV 3D printer (Asiga, Australia) operating at irradiation of 385 nm, with an *X* – *Y* axis resolution of 35 μm. The printed files were created using Autodesk Inventor Professional 2023 software as Computer-Aided Design (CAD) files. The files were designed as arrays of square pillars with varying width (*x*), spacing (*y*), and height (*z*). These designs were converted to Standard Tessellation Language (STL), typically known as stereolithography files. The printing process was done by slicing the predesigned model into 2D slices using the Asiga Max X35 UV software. The printing of the initial layer, also known as the burn-in layer, was done under a light intensity of 26 mW cm^–2^, and an exposure time of 11 s. Tables S1 and S2 present all printing parameters for the subsequent printed layers, including exposure time and light intensity. It should be noted that the inherent feature of DLP printing is the formation of individual layers at the *Z* plan, which brings structuring to that axis. For SUA-2, the light intensity was 0.5 mW cm^–2^ with 25–60 s exposure, depending on the pillar’s dimensions. To remove any uncured material trapped within the voids of the printed pillars, the structures were immersed in acetone for ∼5 min. Finally, the printed surfaces were dried by air pressure to ensure proper cleaning and underwent 15 min of postcuring at 365 nm (2.3 mW cm^–2^ intensity).

### 2.4 Contact Angle Measurements

The contact angle measurements were performed by contact angle meter (OCA 15 DataPhysics) using a sessile drop method. Each measurement involved depositing an 8 μL droplet of distilled water onto the surface of the printed pillars. Two replicates were printed for each pillar dimension, and four measurements were taken at random locations across the two samples. The reported values are the average of these measurements.

### 2.5 Rolling Angle Measurements

Rolling angle measurements were carried out utilizing a tiltable plate (Figure S2). A 20 μL droplet of distilled water was deposited onto the surface of the printed object using a manual pipet (20–200 μL). The object was first mounted on a horizontally tiltable plate, and the plate was gradually tilted from the horizontal position until an angle at which the droplet slides off the surface, which is defined as the ‘rolling angle’ (accuracy of approximately 0.5°). Four measurements were taken for two printed samples at random locations. The reported values are the average of these measurements.

### 2.6 Contact Angle Hysteresis Measurements

The measurements of the contact angle hysteresis were performed using OCA 15 DataPhysics by the sessile drop (needle in) method. To measure the advancing and receding contact angles, a 10 μL drop of distilled water was deposited onto the surface of the pillars at a rate of 0.5 μL sec^–1^. Subsequently, the advancing and receding contact angles for three printed surfaces were recorded. The reported value is the average of the measurements.

### 2.7 Durability Tests

The durability tests were divided into 3 categories: stability of the object over a long-term period, stability after 10 days of immersion in distilled water, and stability after 10 days in a 40 °C oven. For the long-term stability test, a printed structure with pillar dimensions of *x* = 70 μm, *y* = 90 μm, and *z* = 250 μm was kept under ambient conditions (room temperature ∼25 °C, ∼ 50% humidity, and room lighting) for a year, and the contact and rolling angles were measured. For the water stability test and the stability test under 40 °C, a printed surface that has aged for six months with the same pillar dimension (*x* = 70 μm, *y* = 90 μm, and *z* = 250 μm) was kept in distilled water or a 40 °C oven, for 10 days, and the contact and rolling angles were measured before and after 3, 5, 7, and 10 days from the exposure to different conditions.

### 2.8 Abrasion Tests

The resistance of the printed objects was evaluated using a sandpaper abrasion test.^52^ A sample based on SUA-1 ink with pillars dimensions of *x* = 70 μm, *y* = 250 μm, and *z* = 250 μm was placed against sandpaper (2000 grit size), with a 200 g weight applied on top. The object was moved linearly over a distance of ∼10 cm, defined as one cycle. After each cycle, the object was placed on a table tilted at 7°, and the rolling off of 20 μL water droplets was tested.

### 2.9 Mechanical Testing

Standard five dogbone models for tensile testing were printed with dimensions of 15 × 5 × 3 mm. Each printed model’s length, width, and thickness were measured at three different areas, and the reported values are the averages. Mechanical testing was conducted using an Instron Universal Testing machine (model 4500, Instron, USA) equipped with a 500N load cell, at a rate of 10 mm min^–1^. The reported value is the average of the five printed models. Young’s modulus was calculated using the slope of the linear portion of the curve, corresponding to the material’s elastic region.

### 2.10 Elongation Measurements

A custom-made elongation device enabled the measurement of the contact angle while stretching the printed sample (Figure S3). The edges of the object were clamped onto the device, and it was gradually stretched up to the required length. The elongation percentage was determined using an integrated scale. Subsequently, the contact and rolling angles of the elongated surface were measured.

### 2.11 Micropillar Distance Measurements under Elongation

The distances between the micropillars were measured using light microscopy (Dino-Lite Edge 3.0) and analyzed through image analysis software (ImageJ). The surface with the dimensional pillars was imaged from the side during stretching using light microscopy (Figure S4). The images were digitally magnified based on the specified scale, and the spacing between the pillars was measured at five random locations across the model. The reported values represent the average of these measurements.

### 2.12 Reversibility Tests

A printed structure with pillars dimensions of *x* = 70 μm, *y* = 90 μm, and *z* = 250 μm was elongated to 0% and 100% in cycles. During the first and second cycles, the contact and rolling angles were recorded at 0% and 100% elongation. Following this, the structure underwent 50 cycles of elongation from 0% to 100%. In the 53rd cycle, the contact and rolling angles were measured again at 0% and 100% elongation.

### 2.13 Morphology Analysis

The surface morphology was analyzed using a profilometer (Dektak XT, BRUKER). Surface roughness was measured by scanning a length of 500 μm over a duration of 60 s, employing the hills and valleys method. The scan resolution was 0.027 μm. Each surface morphology was measured three times, and the reported value represents the average of these measurements.

### 2.14 Scanning Electron Microscopy

The images of the 3D-printed pillars were taken using scanning electron microscopy (XHR-SEM) XHR Magellan 400 L. A layer of iridium coating was applied to the surfaces to enhance surface conductivity and optimize the SEM imaging. The photographs were acquired using an accelerating voltage of 2.0 kV, a current of 6.3 pA, and a magnification of 150x.

## 3 Results and Discussion

**Figure 1:**
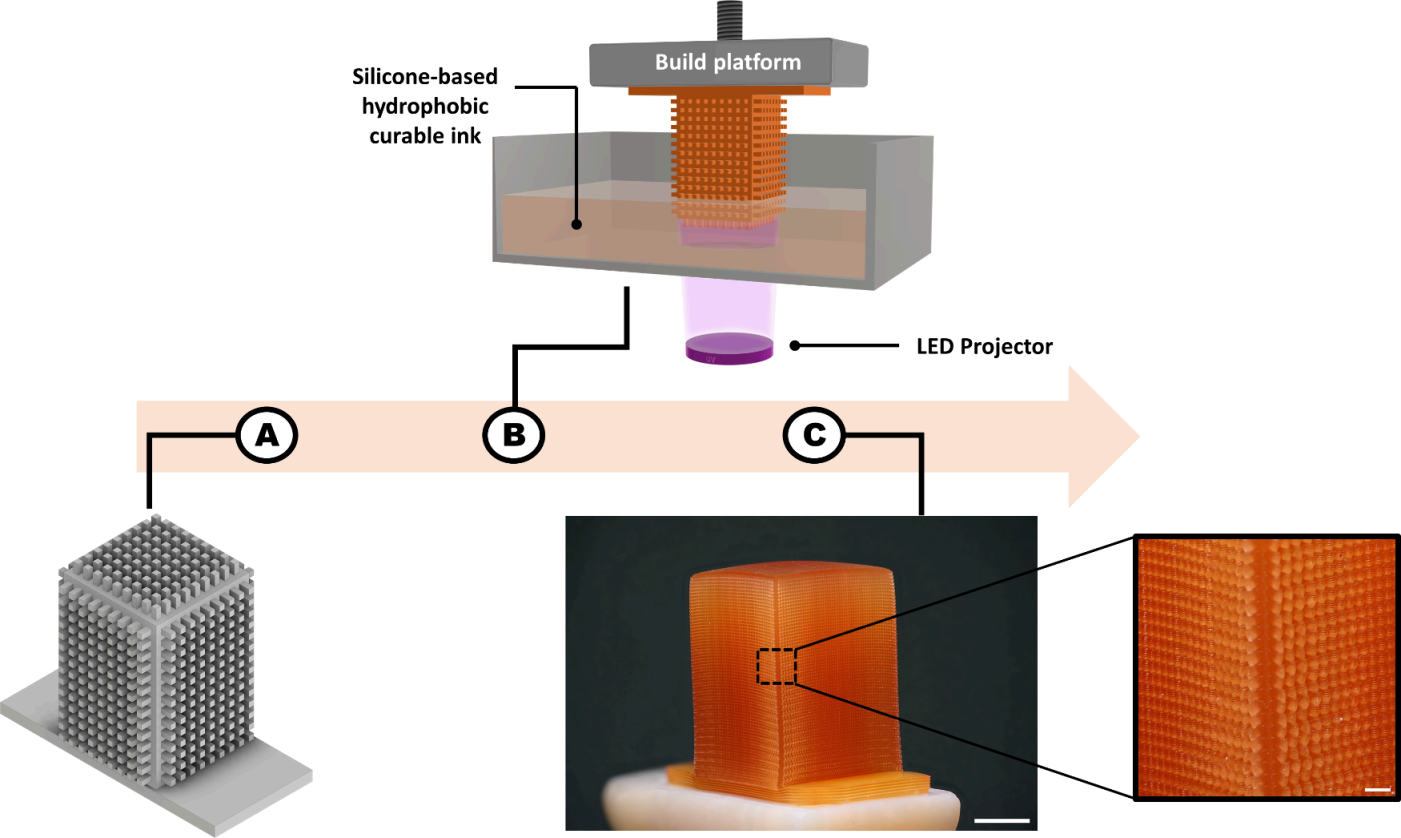
Schematic illustration of the printing process. (A) CAD image of the rectangular cuboid with structural pillars. (B) Schematic fabrication process by DLP. (C) Image of the printed rectangular cuboid with structural pillars (scale bar 5 mm), with magnification of the structural pillars (scale bar 1 mm).

Superhydrophobic structures were fabricated using the DLP method, which is based on localized photopolymerization within a vat by UV light irradiation of 2D layers, according to a predesign CAD file. The process is repeated many times to gradually create the 3D object. Due to the high printing resolution (up to 35 μm in the *x*-*y* plane) of the DLP printer, high accuracy and precision can be achieved. Figure 1 presents schematically the fabrication process, which results in a printed rectangular cuboid with structural pillars that impart the superhydrophobicity. The photocurable formulations contain monomers, photoinitiator, and dispersed hydrophobically modified silica particles. The polymerizable materials are composed of difunctional aliphatic silicone urethane acrylate oligomer (SUA-1), and lauryl acrylate and isobornyl acrylate (IBOA) were used as reactive diluents.

### 3.1 Optimization of the Pillar Design and Ink Formulation for Achieving Superhydrophobicity

Cassie–Baxter state-based superhydrophobicity is highly dependent on the structural design of the surface, which enables retaining air voids and is achieved here by printed pillars. In a comparative substrate, a flat printed surface lacking both pillars and hydrophobic particles has a contact angle of 110° ± 2°, while a printed flat surface with the embedded particles has a contact angle of 117° ± 1°; in both cases, the water drops failed to slide off.

**Figure 2:**
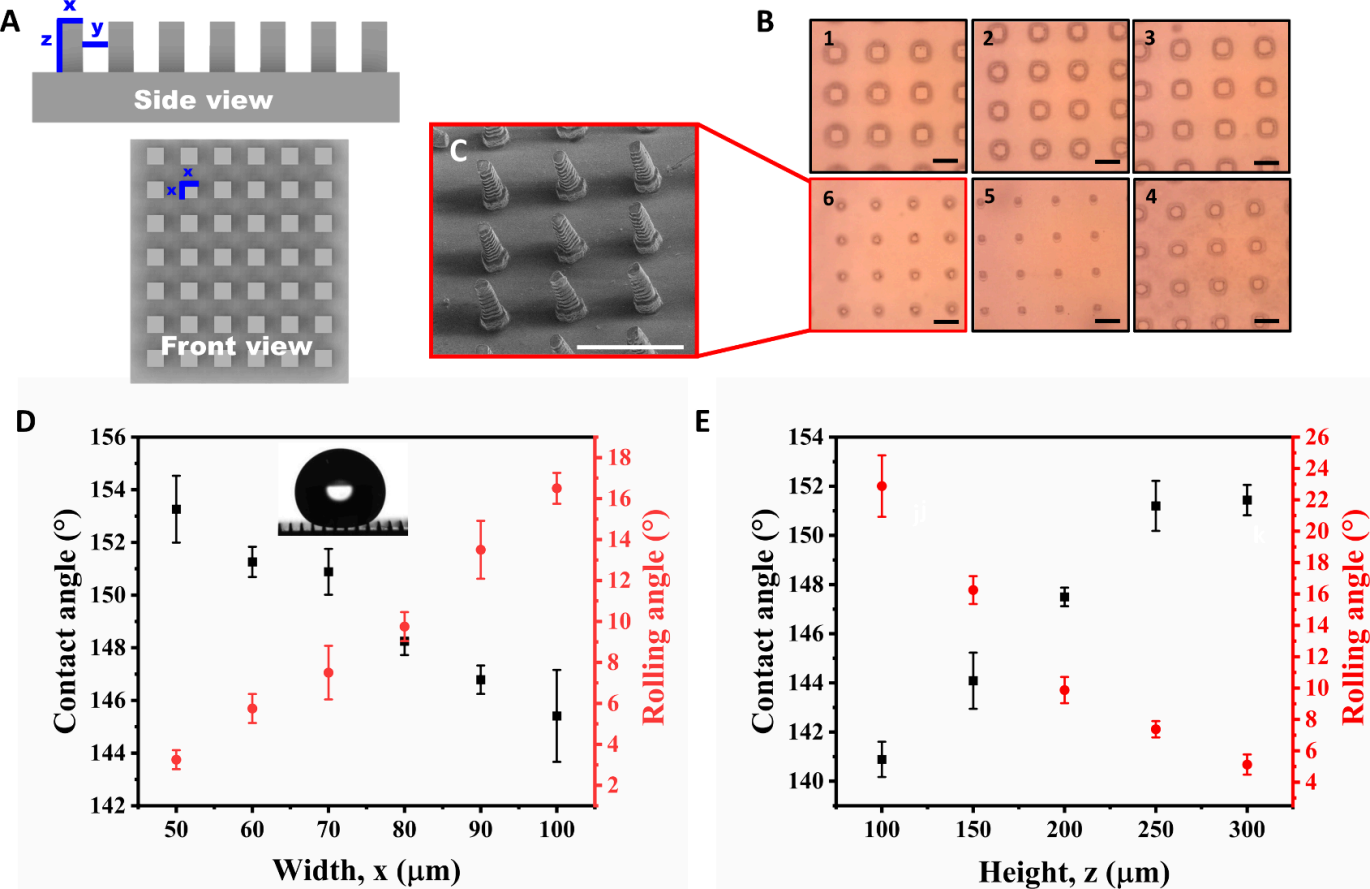
Design optimization. (A) Illustration of the pillar’s dimensions. (B) Light microscopy images of printed surfaces with structural pillars characterized by consistent interpillar spacing (*y* = 250 μm) and height (*z* = 250 μm), alongside varying pillar’s width (*x*). *x* = 100 μm (1), *x* = 90 μm (2), *x* = 80 μm (3), *x* = 70 μm (4), *x* = 60 μm (5), and *x* = 50 μm (6), (scale bar 200 μm). (C) SEM photograph of pillars dimension – *x* = 50 μm, *y* = 250 μm, *z* = 250 μm, 70° tilt, (scale bar 400 μm). (D) Contact and rolling angles while varying width (*x*), constant height (*z* = 250 μm), and interpillar spacing (*y* = 250 μm). In the middle: photograph of water drop on top of the pillar’s surface. (E) varying heights (*z*) at constant width (*x* = 70 μm) and interpillar spacing (*y* = 250 μm).

To optimize the surface design, microsize pillars with diverse dimensions (*x*,*y*,*z*) were printed. As illustrated in Figure 2A, the *x* parameter denotes the pillar’s width, *y* represents the interpillar spacing, and *z* signifies the height of the pillars. By altering the design of the pillar’s width (*x*), interpillar spacing (*y*), and pillar height (*z*), it is possible to attain different wetting properties and achieve superhydrophobicity (water contact angle >150° and water rolling angle <10°). Figure 2B demonstrates the variation in the pillar’s width (*x*), and Figure 2C presents an SEM image of individual pillars, indicating an additional surface roughness resulting from the thickness of the printing layers. The observed layers (Figure S5) are not planned by the design of the pillars, they result from the printing process itself, in which the printer’s parameter for layer thickness was set to 25 μm. Figure S6 shows the variation in the interpillar spacing (*y*) and height (*z*), respectively. The surface with the pillars results in water droplets residing in a Cassie–Baxter state (Figure 2D, inset image). The contact and rolling angle for the surfaces having different dimensions of pillars were measured (Figures 2D, 2E, and S7) to determine the effect of the pillar’s width (*x*), height (*z*), and interpillar spacing (*y*). It was found that increasing the pillar’s width caused a decrease in the contact angle and an increase in the rolling angle, while the opposite effect was seen when increasing the pillar’s height or spacing (Figures 2D and S7). For instance, with a pillar width of *x* = 100 μm, the measured contact angle is 145.4° ± 1.7° and the rolling angle is 16.5° ± 0.8°. When the width decreases to *x* = 50 μm, the contact angle increases to 153.3° ± 1.3°, and the rolling angle decreases to 3.3° ± 0.5°. Clearly, the width, height, and spacing dictate the contact and rolling angle (Figures 2D, 2E, and S7). Based on the printing experiments and the wetting results, it was concluded that the optimal dimensions for achieving superhydrophobicity while maintaining a simple and swift printing process are *x* = 70 μm, *y* = 250 μm, and *z* = 250 μm. In those pillar’s dimensions, the contact and rolling angles were 151.0° ± 1.0° and 7.3° ± 0.7°, respectively. Furthermore, analysis of the advancing and receding contact angles of the surface (Figure S8) revealed that the contact angle hysteresis remains under 10°, in agreement with the characteristics expected of a superhydrophobic surface. Moreover, the effect of experimental printing conditions on wettability were evaluated in view of the wetting properties while changing the exposure time during printing (nominal pillars dimensions of *x* = 70 μm, *y* = 250 μm, and *z* = 250 μm) using SUA-1 based ink. Figure S9 shows the effect of exposure time on the pillar dimensions and resulting wetting properties, while other parameters (Table S.1) remained constant, compared to the exposure time reported in the selected system (Figure S9.B) When the exposure time was reduced to 0.25 s (Figure S9.A), the pillars were under-cured, resulting in a smaller size at the *Z* and *X-Y* planes. The measured contact angle was 132° ± 3°, and the water droplet failed to slide off, indicating the surface was not superhydrophobic. Furthermore, as seen, the droplet on top of the pillars sinks in between the pillars, resulting in a Wenzel wetting state instead of Cassie–Baxter. In contrast, when the exposure time was increased to 9 s (Figure S9.C), the pillars were overcured, resulting in larger pillars dimensions, including partial filling of the spacings, thus resembling a flat surface, lacking the roughness necessary to achieve superhydrophobicity. The measured contact angle was 120° ± 3°, with the droplet again failing to slide off.

**Figure 3:**
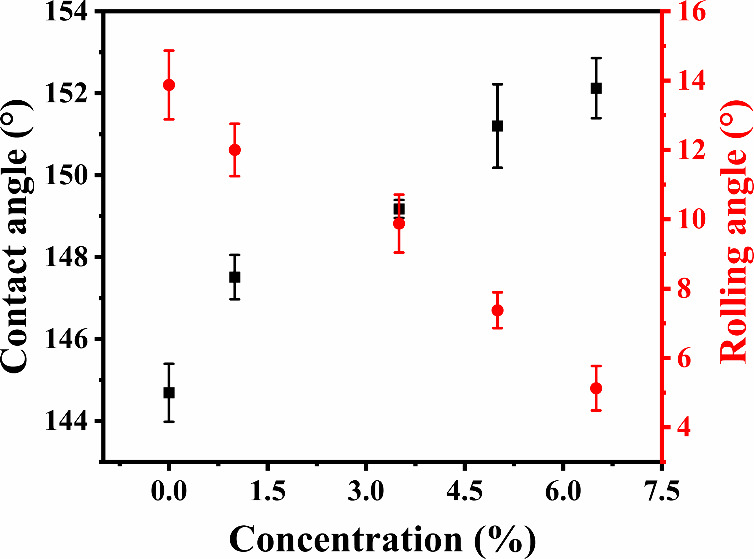
Effect of HFS concentration (wt %) on the contact and rolling angle of a printed surface with constant pillars dimensions: *x* = 70 μm, *y* = 250 μm, *z* = 250 μm.

The effect of the HFS concentration on the superhydrophobicity, as presented in Figure 3, was examined through contact and rolling angle measurements of printed surfaces with structural pillars. The HFS concentration was in the range of 0 wt % to 6.5 wt %. The surfaces were printed with constant pillars dimensions of *x* = 70 μm, *y* = 250 μm, and *z* = 250 μm, as it was denoted as the optimal dimensions for achieving superhydrophobicity, and the contact and rolling angle were measured. In the specified range of HFS concentrations, the printed surfaces exhibited contact angles ranging from 145° to 153° and rolling angles between 14° and 5°. Surfaces without HFS (0 wt %) failed to exhibit superhydrophobic properties, showing a water contact angle of 144.7° ± 0.7° and a rolling angle of 14.0° ± 1.0°. Nonetheless, it still displayed large contact angles and minimal rolling angles, therefore underscoring the primary importance of structural design in achieving the Cassie–Baxter state. Figure 3 shows that the hydrophobicity increases with the increase in HFS particle concentration. The printed surface with a concentration above 5 wt % becomes superhydrophobic. It should be noted that above 5% particles, the ink becomes too viscous for printing. Consequently, 5 wt % of HFS remained constant in the subsequent experiments.

To evaluate the surface roughness caused by the HFS particles, a profilometer test was performed on a flat printed surface without particles, and with 5 wt % of HFS particles (Figure S10). The measurements indicate that objects with HFS particles exhibited an average roughness of 450 nm ±54 nm. In comparison, those without particles (polymer only) have a much lower average roughness of 28 nm ±2 nm. This highlights the hierarchical structure obtained by both surface structuring by printing which is inherent to the layer-by-layer DLP printing technology, and the surface roughness resulting due to the presence of particles. It should be noted that the effective particles are only those present in the vicinity of the surface, while most of them are located within the object, as seen by SEM imaging of a cross-section of the printed object (Figure S11).

### 3.2 Durability

The performance of the superhydrophobic printed objects with fixed pillar dimensions of *x* = 70 μm, *y* = 250 μm, and *z* = 250 μm was evaluated by subjecting it to various conditions and measuring the contact and rolling angles. To assess the long-term effect on superhydrophobicity, an object was exposed to ambient conditions for a year. The contact angle and rolling angle after this period were 151° ± 1° and 7° ± 1°, respectively, indicating that the superhydrophobicity was preserved over time. To examine the impact of water immersion on superhydrophobicity, a 6-month-old object was submerged in distilled water for 10 days. The initial contact angle was 151.2° ± 0.9°, and the rolling angle was 6.4° ± 0.5°. Measurements of the contact and rolling angles were taken on the third, fifth, seventh, and 10th days of immersion. After 10 days in distilled water, the contact and rolling angles remained nearly unchanged, 151.0° ± 0.5° and 7.6° ± 1.5°, respectively, demonstrating sustained superhydrophobicity. To evaluate the impact of elevated temperature on superhydrophobicity, a separate test was conducted where a 6-month-old object was exposed to 40 °C in an oven for 10 days. Initially, the contact and rolling angles were measured at 151.2° ± 0.7° and 6.8° ± 1.1°, respectively. Subsequent measurements were taken on the third, fifth, seventh, and 10th days of exposure. After 10 days at 40 °C, the contact and rolling angles remained nearly the same, 150.9° ± 0.5° and 6.2° ± 0.4°, respectively, demonstrating that the superhydrophobic properties were retained. The durability was further evaluated by an abrasion test. Video S1 shows the abrasion test of the printed object with pillar dimensions of *x* = 70 μm, *y* = 250 μm, and *z* = 250 μm. It can be seen that after the first abrasion cycle, the object retains its superhydrophobicity, with all water droplets successfully rolling off at a 7° tilt. This behavior is maintained until the seventh cycle, indicating good resistance. However, after 8 abrasion cycles, some of the water droplets no longer slide off throughout the whole surface, since some of the pillars at different locations were either removed or lost their height. Further optimization of the ink composition will be required in the future, mainly by increasing the degree of cross-linking of the polymer.

### 3.3 Self-Cleaning, Water-Repellency, and Buoyancy

**Figure 4:**
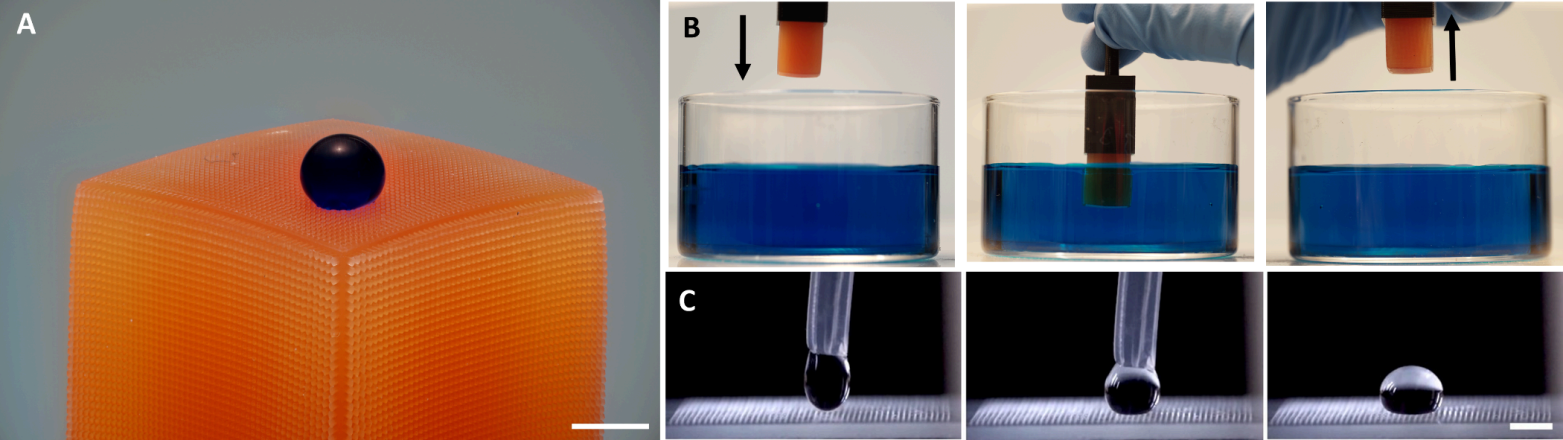
Self-cleaning and water repellency. (A) Photograph of a 10 μL dyed water drop on top of a rectangular cuboid with structural pillars (scale bar 5 mm). (B) Immersion of the printed tube with the structural pillars into methylene blue dye solution, Left–prior to immersion, middle–during immersion, right–after immersion. (C) Photographs of placing a water drop on top of a surface with structural pillars, right and middle - during the process. Left–after the process (scale bar 2 mm).

To assess the capability to remain self-cleaned, a surface with pillars containing 5 wt % particles and the optimal dimensions was used for a quantitative experiment (Video S2, Figure 4C). Ground coffee particles were placed onto the surface, and it was found that they could be easily rinsed off by a drop of water. The extreme water repellency of the surface was also evaluated compared to a flat surface lacking the pillars, as demonstrated in Video S3 and Video S4. It can be seen that a relatively large volume (∼20 μL) of the water droplet is required to overcome this repellent force and establish contact with the surface. Another aspect of the superhydrophobic structure is the ability to stay dry; this was examined through immersion of a printed rectangular cuboid with pillars into an aqueous dye solution (Figure 4B, Video S5). The printed rectangular cuboid stayed dry and clean after the immersion, showing a nonwetting behavior attributed to superhydrophobicity.

**Figure 5:**
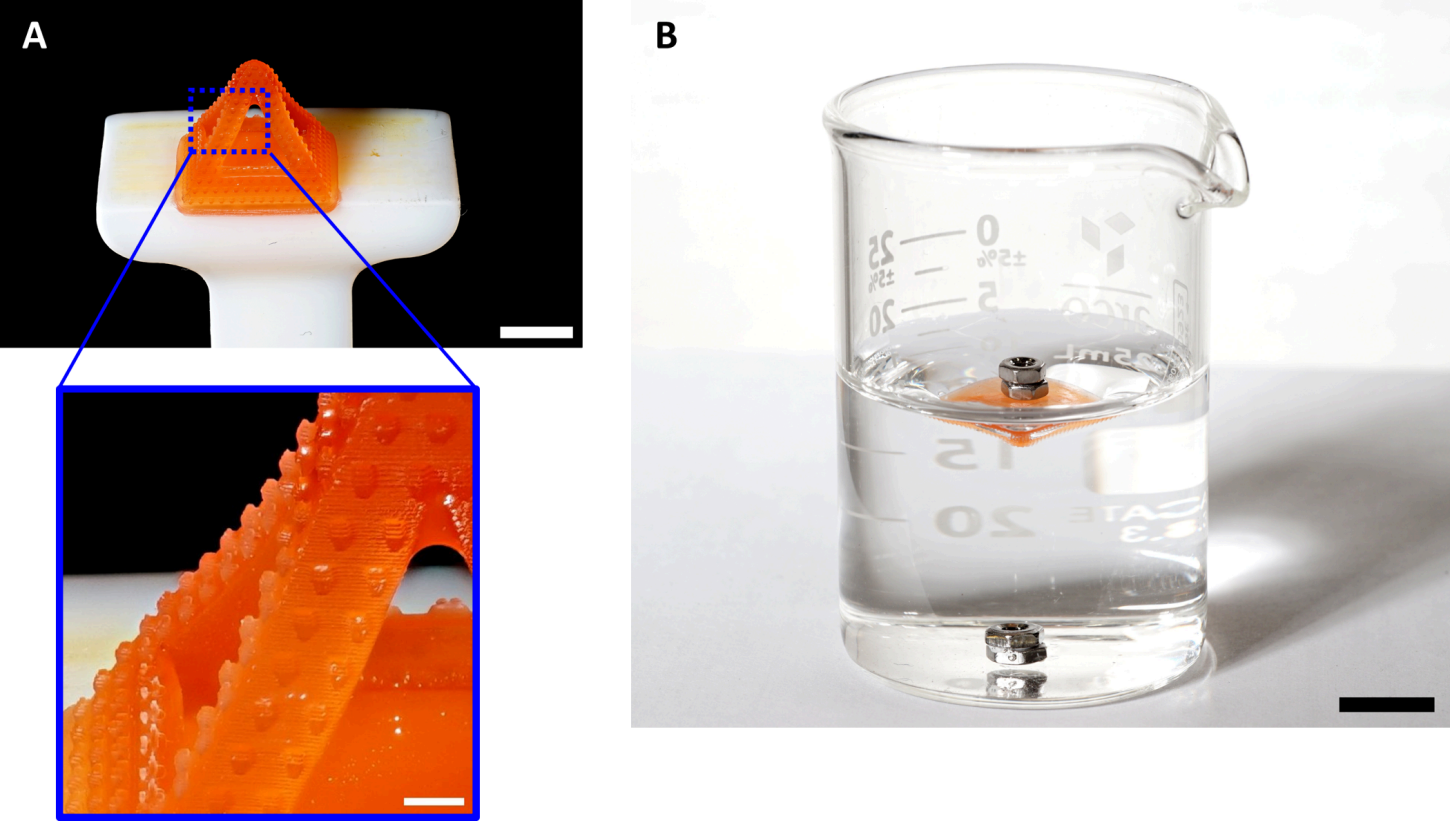
Buoyancy behavior. (A) Photograph of a printed hollow pyramid with structural pillars (scale bar 5 mm), with magnification (scale bar 1 mm). (B) Photograph of the floating bolts on top of the printed hollow pyramid with the structural pillar and the bolts, which sink immediately at the bottom (scale bar 10 mm).

An additional feature that can be observed in superhydrophobic structures is their capacity to float in water. To address this property, a 3D hollow pyramid with structural pillars was printed (Figure 5A). It was immersed in distilled water and demonstrated buoyancy under a weight of 237 mg, ∼ 240% from the pyramid weight (two small bolts, Figure 5B). It should be noted that the two bolts immediately sank when not supported by the pyramid.

### 3.4 Tunable Superhydrophobicity via Stretching

**Figure 6:**
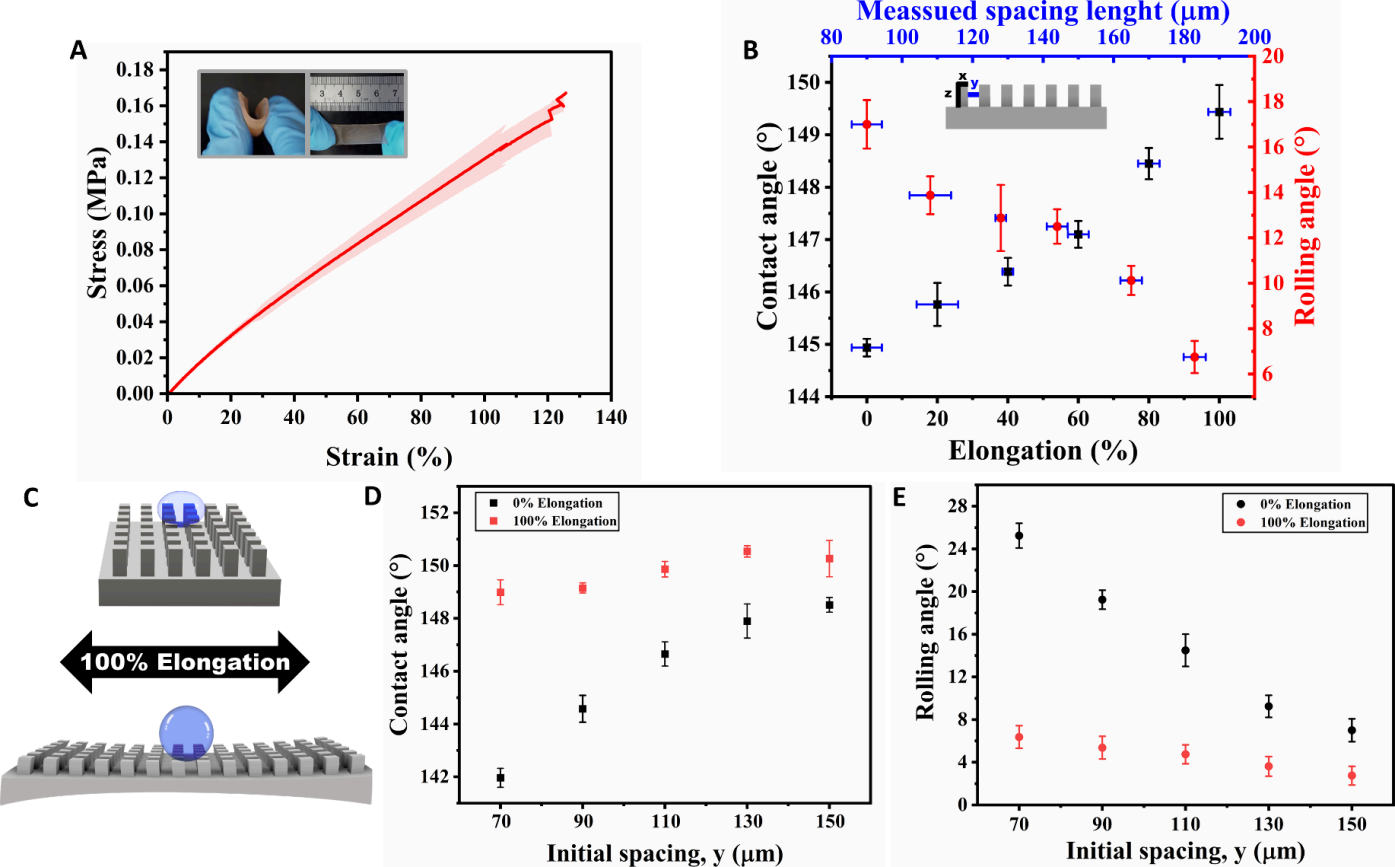
Tuned superhydrophobicity by stretching the printed surface. (A) Tensile test of the stretchable silicone-based (SUA-2) ink. The inset images present the flexibility and stretchability of the surface. (B) Contact and rolling angles in varying elongation percentages of the surface with fixed dimensions: *x* = 70 μm, *y* = 90 μm, *z* = 250 μm. (C) Schematic illustration of the surface and water drop before and after 100% elongation. (D) Contact and (E) Rolling angle measurements of printed surfaces with different interpillar spacing dimensions while maintaining a constant width (*x* = 70 μm) and height (*z* = 250 μm) during 0% and 100% elongation.

To investigate the effect of stretching the printed surfaces on the wetting properties, the elongation of the printed models was first tested (Figure S12). It was found that the ink formulation used up to this point, based on SUA-1 oligomers, exhibits ∼80% elongation at break and Young’s modulus of 0.74 MPa. Furthermore, it was found that the addition of the HFS particles is responsible for the enforcement of the printed polymer (Figure S13), increasing the strength from 0.32 MPA for a sample printed without particles to 0.52 MPa for a sample containing 5 wt % particles (the maximal concentration that still enabled printing), respectively. To extend the elongation at break, a similar SUA oligomer denoted as SUA-2 was used. Figure 6A shows that the maximum elongation at the break of SUA-2 based ink exceeds 120% with Young’s modulus of 0.18 MPa. Given that both SUA oligomers stem from the same polymeric group and the ink compositions are nearly identical, the findings to this point can be extrapolated to validate the results for the ink formulation based on SUA-2. The high stretchability and flexibility (Video S6 and the inserted photographs in Figure 6A) can give rise to the concept of tunable wetting upon stretching, which relies on changing the spacings between the pillars by mechanical stretching.

As a proof of concept, a surface with pillars dimensions of *x* = 70 μm, *y* = 90 μm, and *z* = 250 μm, was printed and was stretched up to various elongation levels while measuring the contact and rolling angle in each level, and the interpillar spacings. Figure 6B presents the dependence of contact and rolling angles on the elongation and, consequently, on the pillars’ spacings. Initially, prior to elongation, the surface is not superhydrophobic, exhibiting rolling and contact angles of 17° ± 1.1° and 144.9° ± 0.2°, respectively (Video S7). Once the elongation reaches 100%, the rolling angle decreases to 6.8° ± 0.7°, and the contact angle increases to 149.4° ± 0.5°, indicating the attainment of superhydrophobicity. Stretching the printed surface causes an increase in the interpillar spacing, resulting in larger voids of trapped air, which induces superhydrophobicity. When the surface undergoes elongation, the drop slides off when the table is tilted to 6° (Video S8). However, when the surface is not elongated and placed on a 6° tilted table, the drop fails to slide off the surface (Video S9). To examine the wetting reversibility, a printed structure (pillars dimensions of *x* = 70 μm, *y* = 90 μm, *z* = 250 μm) was elongated from 0% to 100% for 53 cycles, and the contact and rolling angles were measured in the first, second and 53 cycles. In the first cycle, before the elongation, the contact and rolling angles were 145.2° ± 0.6° and 17.3° ± 1.5° respectively, and at 100% elongation, they were 148.4° ± 0.3° and 7.5 ± 1.3° respectively. At the 53rd cycle, similar results were obtained; before the elongation, the contact and rolling angles were 144.3° ± 1.1° and 19.5 ± 1.7° respectively, and at 100% elongation, they were 148.2° ± 0.3° and 9.5 ± 1.0° respectively. These results indicate a good reversibility of the wetting.

To further investigate this trend, surfaces with pillars with a width of *x* = 70 μm and height of *z* = 250 μm with various interpillar spacing (y) were printed and tested for contact and rolling angles at 0% elongation and 100% elongation, as schematically illustrated in Figure 6C. It is evident from Figure 6D and Figure 6E that at interpillar spacing resembling the pillar’s width, such as 70 and 90 μm, there is a significant change in the contact and the rolling angles, resulting in surfaces becoming superhydrophobic upon stretching to 100%. The most significant impact of stretching is observed when the initial spacing between the pillars is relatively small: when the interpillar spacing is 70 μm, and the surface is not stretched, the contact angle is 141.9° ± 0.4°, and the rolling angle is 25.3° ± 1.2°, indicating that the surface is not superhydrophobic. However, upon 100% elongation of the surface with 70 μm interpillar spacing, it becomes superhydrophobic, with a contact angle of 149.0° ± 0.5° and a rolling angle of 6.4° ± 1.1°. Notably, when the interpillar spacing was 150 μm, the contact and rolling angles did not change dramatically upon stretching: 148.5° ± 0.3° and 7.0° ± 1.1°, respectively, without stretching, and 150.3° ± 0.7° and 2.8° ± 0.9°, respectively, upon 100% stretching. This could be attributed to the sufficient air voids, which are present even on the unstretched surface.

## 4 Conclusions

Here, we presented an approach for fabricating soft superhydrophobic 3D structures using digital light printing. The DLP approach is a simple and rapid method that offers the creation of complex superhydrophobic geometries in one-step fabrication at high resolution. We formulated new photocurable ink compositions composed of soft silicone urethane acrylates and dispersed hydrophobic silica particles. It was found that the ink composition without the hydrophobic particles is hydrophobic (contact angle 110° due to the selected monomers, compared to our previous work.^52^ 3D micropillars structures were designed and printed to impart superhydrophobicity due to the Cassie–Baxter state. Unlike other superhydrophobic surfaces that rely on structural designs to induce the Cassie–Baxter state, this work leverages material properties and a precise additive manufacturing process based on stereolithography by low-cost printers to control wetting behavior. In other reports, such as by Wang et al.^64^ micronanostructures with a dual-energy barrier design were fabricated by ultrafast laser ablation, resulting in an array of open microcones. The resulting structure introduced a double energy barrier, stabilizing the Cassie–Baxter state. In our current study, 3D structures with micropillars resulted in a Cassie–Baxter wetting state, by using new printing compositions containing hydrophobic particles, in a single fabrication step. Combining the structuring of pillars, with the layered structure inherent to the DLP layer-by-layer printing technology, with the silica particles, results in hierarchical 3D structures that impart superhydrophobicity. It was found that the optimal pillars dimensions for achieving superhydrophobicity and maintaining a comfortable printing process were *x* = 70 μm, *y* = 250 μm, and *z* = 250 μm. The 3D structures exhibited self-cleaning, water repellency, and buoyancy properties. Additionally, the superhydrophobicity properties are maintained for a year under standard room conditions and 10 days after exposure to water or high temperature. Tuned superhydrophobicity was achieved by stretching the printed structures, thus causing changes in the interspacing lengths. It was found that under 100% elongation, a surface with pillars dimensions of *x* = 70 μm, *y* = 90 μm, *z* = 250 μm, which initially is not superhydrophobic, becomes superhydrophobic upon stretching to 100%; The wetting properties are reversible. The stretchable structures with tuned superhydrophobicity can have potential applications in soft robotics, wearable and stretchable electronic devices, and medical equipment. We expect that this concept of controlling wetting properties can be applied to various elastomers, and other hydrophobic embedded materials, such as hydrophobic nanowires enabling better stretchability of the structures.

## Supporting Information Available

The Supporting Information provides additional data and experimental evaluations: Figure S1: Chemical structure of reactants and polymerization reaction. Tables S1 & S2: Printing parameters. Figures S2–S13: rolling angle device (S2); elongation device (S3); light microscopy images under elongation (S4); SEM image of individual pillar (S5); light microscopy images with varying *y* and *z* dimensions (S6); contact and rolling angle vs varying *y* dimension (S7); contact angle hysteresis (S8); effect of printing parameters on pillar dimensions and wetting (S9); roughness profiles (S10); SEM image of cross-section (S11); tensile testing (S12); tensile testing with varying percentage of HFS particles (S13) (PDF)
Abrasion test (Video S1 (MPG)); self-cleaning (Video S2 (MPG)); water repellency (Videos S3 and S4 (MPG) (MPG)); nonwetting (Video S5 (MPG)); flexibility (Video S6 (MPG)); sliding angles (Videos S7–S9 (MPG), (MPG), (MPG))

## Supplementary Material

am4c14238_si_001.pdf

am4c14238_si_002.mpg

am4c14238_si_003.mpg

am4c14238_si_004.mpg

am4c14238_si_005.mpg

am4c14238_si_006.mpg

am4c14238_si_007.mpg

am4c14238_si_008.mpg

am4c14238_si_009.mpg

am4c14238_si_010.mpg
