# Supplementary material for: Tunable Lotus Leaf Effect by Three-Dimensionally Printed Stretchable Objects
Source: ACS Appl Mater Interfaces. 2024 Nov 6;16(46):64276–86. doi: 10.1021/acsami.4c14238 (PMC11583117; doi:10.1021/acsami.4c14238)
Supplement: Supplementary file 1 — am4c14238_si_001.pdf [file am4c14238_si_001.pdf]

Supporting information

Tunable Lotus Leaf Effect by 3D-printed stretchable objects

Noa Trink[ab] *and Shlomo Magdassi**[ab]

[a] *Institute of Chemistry and The Center for Nanoscience and Nanotechnology, The Hebrew University of Jerusalem, Jerusalem 9190401, Israel.*

[b] *Singapore-HUJ Alliance for Research and Enterprise (SHARE), Smart Grippers for Soft Robotics (SGSR), Campus for Research Excellence and Technological Enterprise (CREATE), Singapore, 138602, Singapore.*

E-mail: magdassi@mail.huji.ac.il

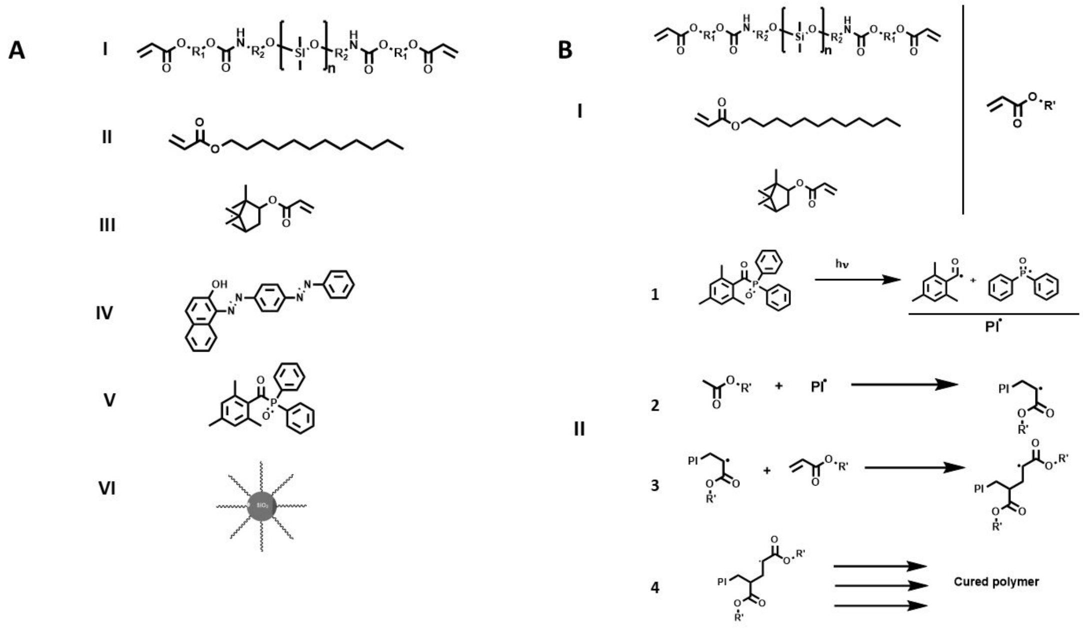

Figure S1. (A) Chemical structure of all reactants and materials (I) Difunctional aliphatic silicone urethane acrylate, (II) Lauryl acrylate, (III) Isobronyl acrylate (IV) SUDAN III (V) TPO, (VI) Fumed silica particles. (B) Photopolymerization mechanism. (I) Chemical structure of the participating monomers. As all monomers are acrylates, they are labeled with the schematic illustration shown. (II) polymerization mechanism; First (1), the photoinitiator dissociates under irradiation, forming radicals. Next (2), these radicals initiate the polymerization by reacting with acrylate’s double bond. Further propagation (3) is obtained by continuous reaction between radicals and acrylates. Finally (4), the radicals terminate, forming the final cross-linked structure. Note that since one of the monomers contains two acrylate groups, the results polymer is cross-linked.

Table S1. Printing parameters for Asiga Max X35 (385nm)

Build Parameters	Burn-in layers	Subsequent layers SUA-1	Subsequent layers SUA-2
Light intensity	27 mW/cm2	25 mW/cm^2	0.5 mW/cm^2
Slice Thickness	0.2 mm	0.025 mm	0.05mm
Separation distance	12 mm	12 mm	12 mm
Separation velocity	1.5 mm/s^2	0.8 mm/s^2	0.8 mm/s^2
Approach velocity	3 mm/s^2	1.5 mm/s^2	1.5 mm/s^2
Approach deceleration	0.5 mm/s^2	0.3 mm/s^2	0.3 mm/s^2
Exposure time	11 sec	1.5 sec	*
Wait time (after exposure)	3 sec	3 sec	3 sec
Wait time (after separation)	5 sec	3 sec	3 sec
Wait time (after approach)	0 sec	0 sec	0 sec
Normal Approach Pressure Limit	100 g/cm^2	100 g/cm^2	100 g/cm^2

*Depending on the inter-pillar spacing: see Table S2

Table S2. Exposure time for SUA-2 Subsequent layers

Inter-pillar spacing-y	Exposure time
70µm	25 sec
90µm	32 sec
110µm	35 sec
130µm	40 sec
150µm	60 sec

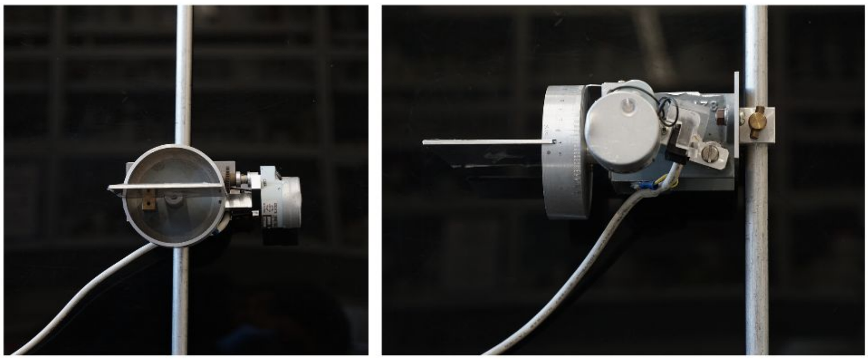

Figure S2. Rolling angle measurements device. Images from different angles.

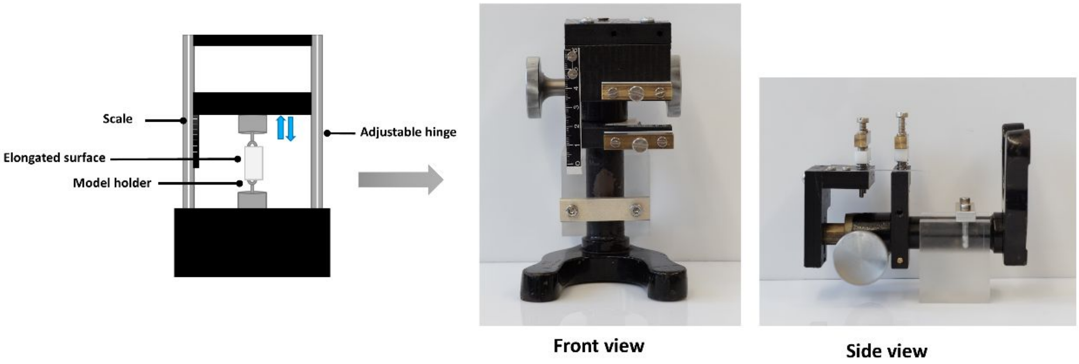

Figure S3. Elongation device. Left- schematic illustration of the elongation process. Right – front and side view images of the device.

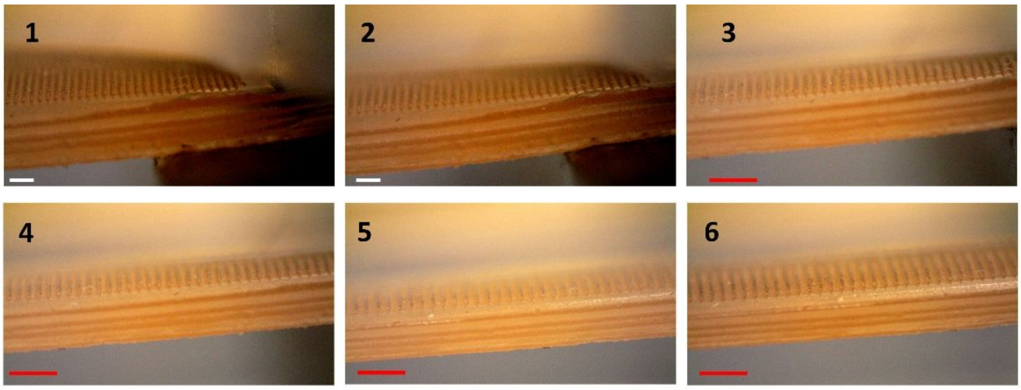

Figure S4. Light microscopy images of printed surfaces with structural pillars under varying degrees of elongation: (1) 0% elongation, (2) 20% elongation, (3) 40% elongation, (4) 60% elongation, (5) 80% elongation, and (6) 100% elongation, (white scale bar 500 µm, red scale bar 1000 µm).

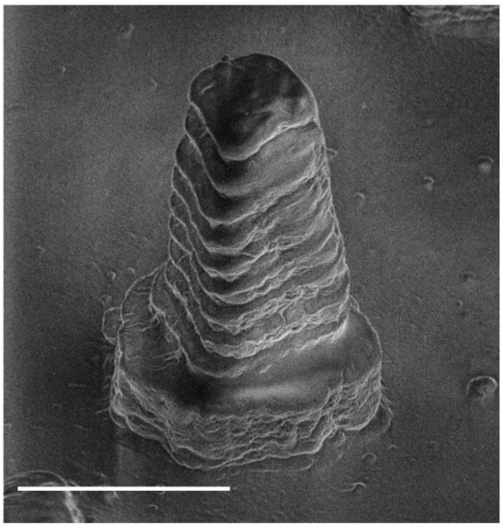

Figure S5. SEM photograph of individual pillars with dimensions of - x = 50 µm, y = 250 µm, z = 250 µm, (scale bar 100 µm).

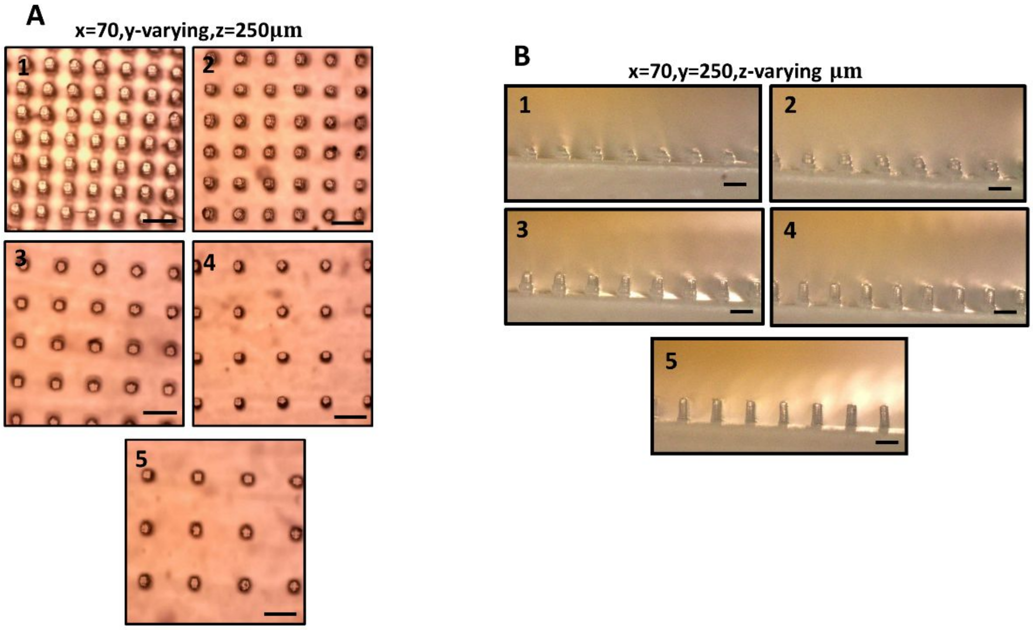

Figure S6. Light microscopy images of printed surfaces with structural pillars characterized by; (A) consistent pillar's width (x = 70 µm) and height (z = 250 µm), alongside varying inter-pillar spacing (y). y = 100 µm (1), y = 150 µm (2), y = 200 µm (3), y = 250 µm (4), and y = 300 µm (5), (scale bar 200 µm). (B) Consistent pillar's (x

= 70 µm) and inter-pillar spacing (y = 250 µm), alongside varying pillar's height (z). z = 100 µm (1), z = 150 µm (2), z = 200 µm (3), z = 250 µm (4), and z = 300 µm (5), (scale bar 200 µm).

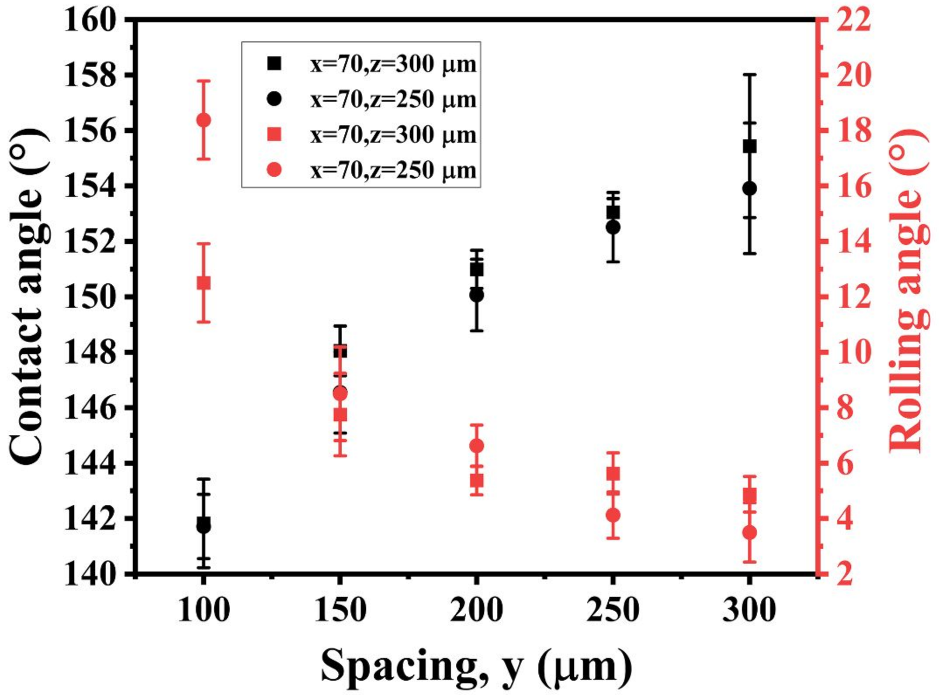

Figure S7. Contact and rolling angle while varying the inter-pillar spacing (y), constant width (x = 70 µm), and height (z = 250 µm or z = 300 µm).

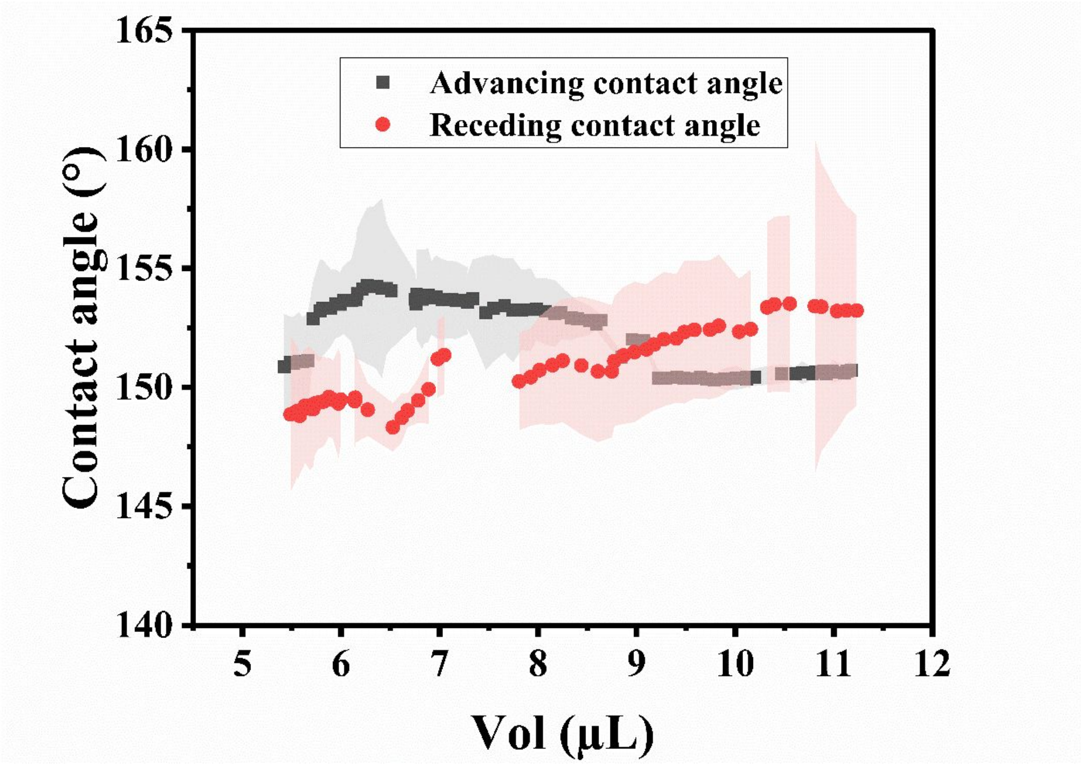

Figure S8. Contact angle hysteresis - advancing and receding contact angle of a printed surface with pillars dimensions: $x = 70$ μm, $y = 250$ μm, $z = 250$ μm.

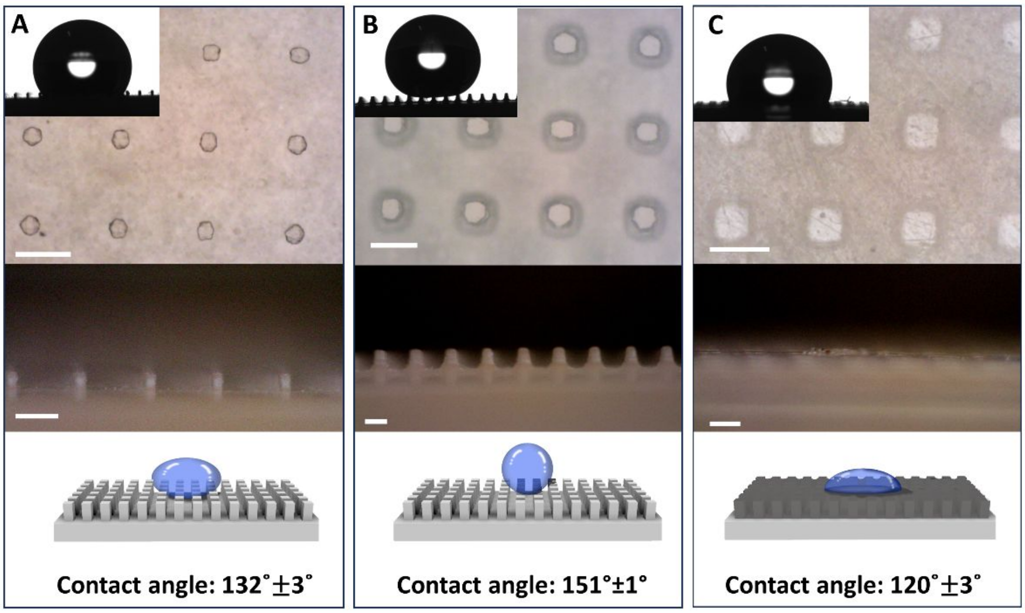

Figure S9. The effect of printing exposure time on the printed pillars dimensions (x = 70 μm, y = 250 μm, z = 250 μm) and wetting properties. **Top** – light microscopy images of the pillars in the *X*-*Y* plane along with an images of the drop on top of the pillars. **Middle** - light microscopy images of the pillars in the *Z* plane. **Bottom** - schematic illustration of the droplet's state on the surface. (A) 0.25 sec exposure time, (B) 1.5 sec exposure time, (C) 9 sec exposure time, (scale bar 200 μm).

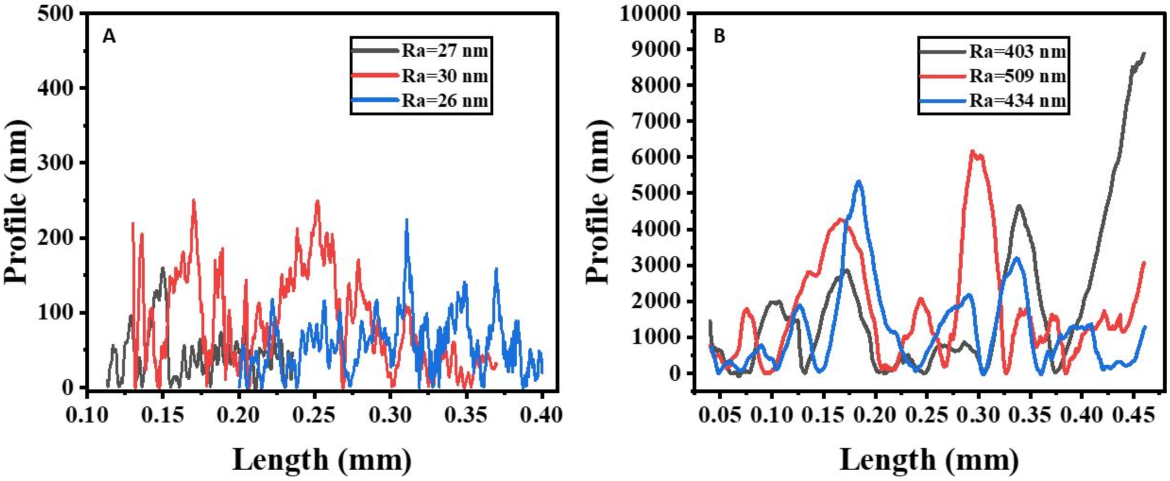

Figure S10. Roughness profiles of smooth printed surfaces using SUA-1-based ink: (A) without HFS particles (0 wt%); (B) with 5 wt% of HFS particles.

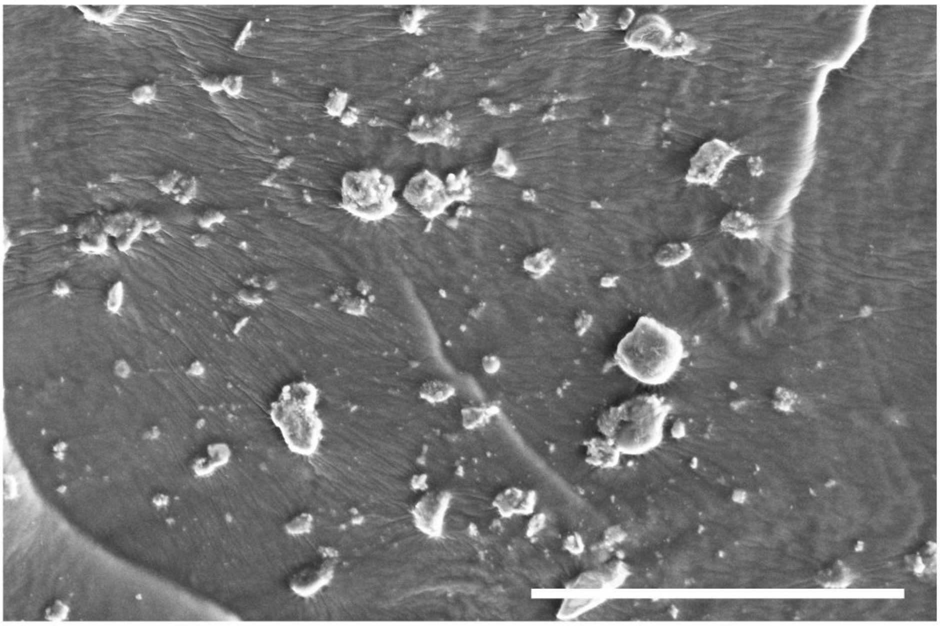

Figure S11. SEM photograph of a cross-section of SUA-1 based printed object with 5 wt% of HFS particles (scale bar 50μm).

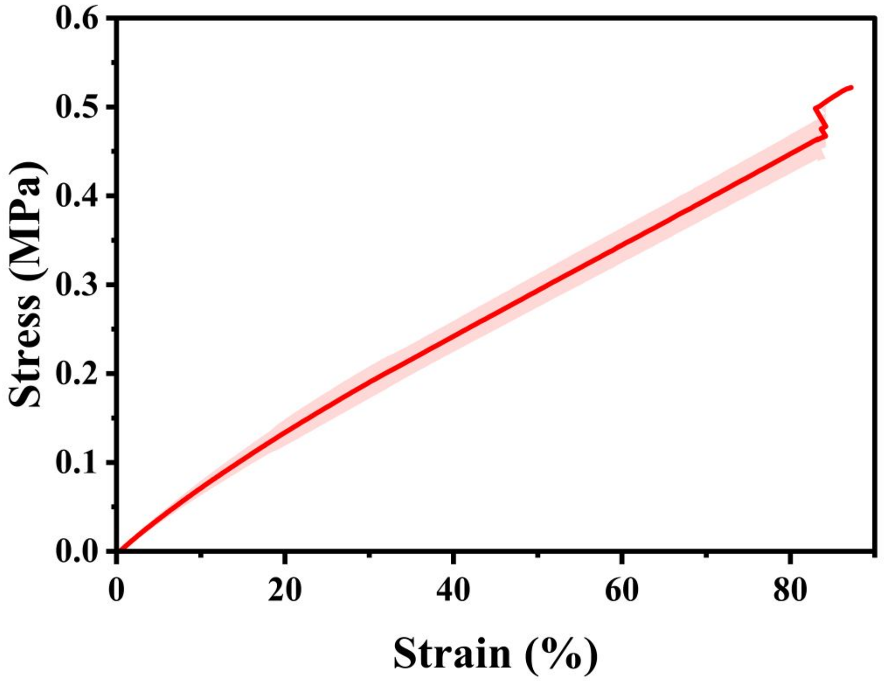

Figure S12. Tensile test of the SUA-1 based ink.

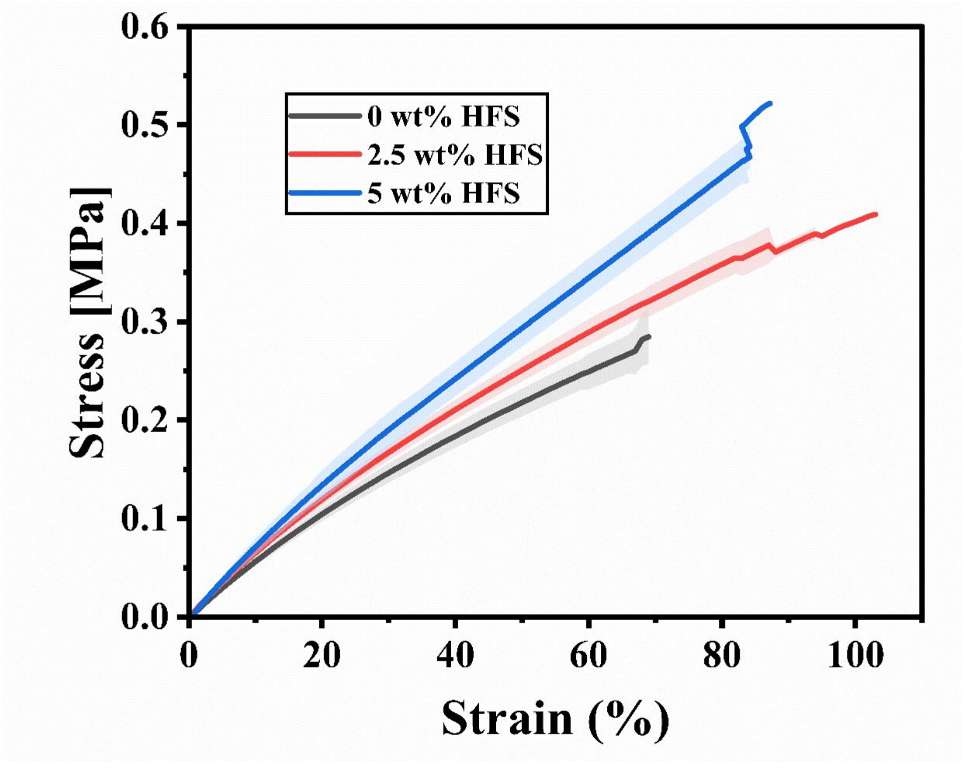

Figure S13. Tensile test of SUA-1 based ink with varying wt% of HFS particles.

Video S1. Abrasion test of a printed surface with pillars dimensions of x = 70 µm, y = 250 µm, z = 250 µm, SUA-1 based ink.

Video S2. Self-cleaning demonstration of a printed surface with structural pillars. The surface was soiled with ground coffee and cleaned by rinsing it with DW.

Video S3. Demonstrator of placing a water drop on a superhydrophobic printed surface with structural pillars.

Video S4. Demonstrator of placing a water drop on a flat printed surface (without structural pillars).

Video S5. Non-wetting demonstration of a printed rectangular cuboid with a structural pillar and a printed rectangular cuboid without a structural pillar. The two printed objects were immersed in an aqueous dye solution.

Video S6. Demonstrator of the flexibility of the surface.

Video S7. Sliding angle demonstration: aqueous dye drops sliding off from a 20º tilted table on top of a 0% elongated printed surface with pillars dimensions of x = 70 µm, y = 90 µm, z = 250 µm.

Video S8. Sliding angle demonstration: aqueous dye drops sliding off from a 6º tilted table on top of a 100% elongated printed surface with pillars dimensions of x = 70 µm, y = 90 µm, z = 250 µm.

Video S9. Sliding angle demonstration: aqueous dye drops failing to slide off from a 6º tilted table on top of a 0% elongated printed surface with pillars dimensions of x = 70 µm, y = 90 µm, z = 250 µm.
